# School bus routing problem in the stochastic and time-dependent transportation network

**DOI:** 10.1371/journal.pone.0202618

**Published:** 2018-08-23

**Authors:** Shichao Sun, Zhengyu Duan, Qi Xu

**Affiliations:** 1 College of Transportation Engineering, Dalian Maritime University, Dalian, Liaoning Province, China; 2 The Key Laboratory of Road and Traffic Engineering, Ministry of Education, Tongji University, Shanghai, China; Beihang University, CHINA

## Abstract

Accidents, bad weathers, traffic congestions, etc. led to the uncertainties of travel times in real-life road networks, which greatly affected the quality of individual’s life and the reliability of transportation system. This paper addressed the school bus routing problem in such a stochastic and time-dependent road environment. Firstly, the problem was set based on a single-school configuration, and the students were picked up at their homes, which was in line with the current situation of school bus systems in China. Thus, it could be regarded as an independent problem of school bus route generation in random dynamic networks, which could be solved as a variant of extended Vehicle Routing Problem. However, due to the fluctuation and uncertainty of link travel times, the arrival time at each stop including the destination was varying. Therefore, the selection of optimal path connecting the current service node with the next one was treated as a sub-problem in this study, where the reliability of travel times in the stochastic and time-varying network was highly concerned by such time-rigid commuters. To this end, a Robust Optimal Schedule Times model with a hard time windows constraint was built to generate a most cost-reliable route for school buses. By the use of Robust Optimization method, it was intended to minimize the worst-case total cost which combined the cost of earlier schedule delays with the disutility of travel times. It was also proved that the proposed model could be converted into solving a conventional problem in deterministic dynamic networks for a reduction of computation complexity, which provided the potential of applying to the practical problems. Finally, the validity of the proposed model and its performance evaluation was analyzed through a small-scale computational instance, where all the link travel times in the simulated network were attributed to both time-varying and stochastic. Then, a mathematical programming solver was used to find the exact optimal solution. The results indicated that the model was valid, and the necessity of considering the stochastic and time-dependent nature of transportation networks was also confirmed in the case study.

## 1 Introduction

For the sake of convenience and safety, the school bus system is usually adopted to deliver students from certain stops to their respective schools in the morning, and return them back after school. Due to the increased demand of the school bus system in the real world and the appearance of routing and scheduling issues during the operation of a vehicle fleet, the School Bus Routing Problem (SBRP) has been widely studied. SBRP can be regarded as a combinatorial optimization problem which have specific assumptions and constraints [1]. It consists of several sub-problems which can be summarized as “bus stop selection problem”, “route generation problem”, “school bell time adjustment problem” and “route scheduling problem” [2]. Each single sub-problem or the combination of them can be converted into solving the existing optimization problems. In most literature, these sub-problems are generally considered separately and sequentially [2]. The “bus stop selection problem” is usually studied to determine the location of bus stops in a given transportation network by taking account of the efficiency of students’ assignment [3]. The “route generation problem” can be regarded as a variant of Vehicle Routing Problem (VRP) [4]. The generated routes in the sub-problem take the bus depot as the origin, and regard the school as the destination, while in reverse after school. It seeks to generate efficient routes for a fleet of school buses to pick up (or deliver) students at each assigned bus stop with objectives such as the number of buses used, total bus travel distance and so on [5, 6]. The sub-problems of “school bell adjustment” and “route scheduling” are usually considered under a multi-school configuration, in which the school buses are shared by multiple schools [7–11]. However, the re-arrangement of school time must be permitted in the “school bell adjustment problem”, as well as the scheduling of the bus fleet for multiple schools should be allowed. Regarding the students from different schools, whether a mixed load being allowed to get on the same bus at the same time is also considered as an important assumption in the sub-problem of “bus route scheduling” [1, 8, 9, 11]. Nevertheless, other than the situation in Korea and other countries in Asia, the school bus fleet in China is mostly operated by the individual school itself. Thus, the school bus system in China is not based on a multi-school configuration, and the regional board of education does not take charge of the operation of the school bus fleet, either. In addition, the opening and closing time of schools are also consistent in a local district, and not allowed to be changed. Therefore, sub-problems of SBRP considering a multi-school shared school bus system is not appropriate for the current situation in China. Regarding the bus stop selection problem, it is also not necessary in most cities of China, since students are commonly picked up at their houses.

Hence, this paper solely addressed the “bus route generation problem” of SBRP under a single-school configuration in the metropolis of China. As an extension of the existing work, the stochastic and time-dependent (STD) nature of link travel times was taken into account in the current paper, other than the common assumptions in most related literature. Accidents, bad weather, traffic congestion, etc. led to the uncertainties of travel times in real-life transportation networks, which greatly affected the quality of individual life and the reliability of transportation system. Thus, the variability in travel times was attributed to both dynamic and uncertainty in the real world, and modelling the SBRP in a stochastic and dynamic network was in line with the reality [12]. In this context, the optimal path problem in STD networks which sought the efficient path connecting the current service node (including the bus depot) to the next one was regarded as a sub-problem of bus route generation. The sub-problem could be categorized into two main aspects: a priori path based formulations and adaptive strategy based formulations, while in this paper the former one should be adopted for school bus routing. However, it is a NP-hard problem where the Bellman’s optimality principle is not applicable, due to the uncertainties and dynamics of travel times. Thus, the assumption related to the attribute of travel time increased the complexity of solving SBRP. Nevertheless, it could be regarded as a general and complex extension to the conventional route generation problem in another type of transportation networks, enriching the literature.

In this paper, the nature of travel times in transportation networks was captured in solving such a problem. The objectives and the time window constraint such as the school bell time was also considered from a different perspective, since the travel time reliability and the possibilities of schedule delays were extremely concerned in STD instances. The paper was organized as follows: in the section 2, a literature review was given which was associated to the route generation problem in SBRP. In the section 3, the STD network was given, notations were defined, and the model was formulated. In the section 4, computational instances and solution approach were designed, and results were analyzed and discussed. In the section 5, conclusions were drawn.

## 2 Literature review

Based on Park and Kim [2], the literature associated to the school bus routes generation based on a single school configuration can be categorized as follows:

- According to the surroundings of service, the solution approaches can be classified depending on whether the service is implemented in rural areas or urban areas. In rural areas, the number of students is small, and students are usually picked up at their homes. Thus, it is not necessary to consider the bus stop selection problem for the case in rural surroundings. While, it is commonly assumed that students in urban areas should walk from their houses to their assigned stops to catch the school bus [2].
- The SBRP can also be classified according to the type of bus fleet: Homogeneous (HO) or Heterogeneous (HT). A heterogeneous fleet of school buses implies that the vehicles may have different characteristics such as various capacities, and cost [9]. While, the problem with a homogeneous fleet of buses assumed that the vehicles were the same. Thus, the problem dealt with a heterogeneous fleet of school buses was the extension of the homogeneous one, and would be more complex.
- The measures for evaluating the performance of school bus services were various. The objectives in the model could be classified from the perspectives of efficiency and effectiveness. The number of buses used (N) and the total bus travel distance or time (TBD) were mostly adopted to assess the performance, as shown in Table 1. A few works also employed total student riding or walking distance (TSD), child’s time loss (TL), and transportation cost (TC) as the measures [1, 2].
- Various constraints were also adopted in the model formulation of SBRP. Vehicle capacity constraint (C), fixed school time window (TW) or time window range (TWR), maximum walking tine (MWT) and maximum riding time (MRT) were the most ones used in the model.

As shown in Table 1, a literature classification could be summarized based on the above practical aspects in the problem. Although a large number of works have been done in the literature of SBRP, regarding the problem scope, however, few studies have discussed the nature of transportation networks itself. The stochastic and time-varying characteristics of link travel times were rarely considered in the SBRP, as far as we knew. Instead, it was usually assumed the travel times in the transportation network were deterministic and static. Neglecting the natural characteristics of travel times in the real world made it hard to apply the research efforts for practice. Thus, considering all have been done in the literature, the current study was based on the practical situation in China, and it extended the existing works in two aspects. Firstly, the travel times in the model were defined as random time-dependent variables, which led to an increase of complexity to solving the problem but was more in line with the reality. In this context, finding the optimal path connecting the current service node to the next one in STD networks was regarded as the sub-problem of SBRP in this paper, which was also a hot topic in the study of shortest/optimal path [21–24]. Secondly, the methodology of Robust Optimization (RO) was employed here to concern the travel time reliability and the possibilities of schedule delays in STD instances.

**Table 1:** Literature classification based on the practical aspects.

Reference	Surrounding of Service	Fleet mix	Objectives	Constraints
[1]	Rural	HT	TC	C, TW, MRT
[5]	Urban	HO	TBD	C
[6]	Rural	HT	TL	C, TWR
[7]	Rural	HO	N, TBD	TWR
[8]	Rural	HT	TBD	C, MRT, TW
[9]	Rural	HT	TSD, TC, LB	C
[10]	Rural	HT	TC	C
[13]	Rural	HO	N, TBD	TWR
[14]	Urban	HO	N	C, MRT, TW
[15]	Urban	HT	N, TBD	C
[16]	Urban	HO	N, TBD, TSD	C, MRT, MWT
[17]	Rural	HO	TSD	C
[18]	Urban	HO	N, TBD	C, MRT, MWT
[19]	Urban	HO	N	C, MRT
[20]	Urban	HO	N, TBD	C

## 3 Model formulation

### 3.1 The STD network

Given a directed network $G=(V,A,T,C_{ab}^{t})$, where *V* is the set of nodes and *A* is the set of links. Link (*a*, *b*) represents the directed link connecting link node *a* with link node *b*. *T* is the time-horizon which could be discretized into a set of time-intervals as *T* = {0, *δ*, 2*δ*…(M − 1)*δ*}, where *δ* is the unit of discretization and *M* is the number of time-intervals.$C_{ab}^{t}$ denotes the travel time of link (*a*, *b*) at time interval *t* (*t* ∈ *T*).

$C_{ab}^{t}$ is defined as:

$$C_{ab}^{t}=R_{ab}^{t}+\tau_{ab}^{t}$$ (1)

$R_{ab}^{t}$ is a fixed travel time value at time interval *t*, and $\tau_{ab}^{t}$ is a random variable with the range of $\left[0,d_{ab}^{t}\right]$, where $d_{ab}^{t}$ is also a fixed value at time interval *t*. Hence $C_{ab}^{t}$ is a random time-dependent variable which takes value within the range of $\left[R_{ab}^{t},R_{ab}^{t}+d_{ab}^{t}\right]$. Moreover, it was assumed that the value range of the link travel time could be directly obtained by analyzing the historical Float Car Data (FCD) or crawling from open-access navigation map dynamically in the practical application. Thus, the best-case and worst-case travel time on the same link could be calibrated in a certain time period, regardless of the type of distribution the variable $\tau_{ab}^{t}$ following.

Furthermore, it is assumed that the network satisfies the Stochastic Consistent Condition (SCC) proposed by Wellman et al. [20]. Namely, for any link (*a*, *b*) ∈ *A* at any time interval *t* ≤ *t′*, and any given time *ξ*, the following inequality holds.

$$\text{Pr}(C_{ab}^{t}+t\leq \xi )\leq \text{Pr}(C_{ab}^{t^{\prime }}+t^{\prime }\leq \xi )$$ (2)

The inequality implies that the probability of arriving at the destination before time *ξ* could not be increased by leaving later. This consistent condition generally holds in the real transportation network, regardless of few overtaking phenomenon.

### 3.2 Notations in the formulation

The notations as well as their definitions in the following formulation were listed in Table 2, according to different types.

**Table 2:** Notations in the formulation.

Type	Symbol	Definition
Constants	*α*_1_, *α*_2_	Weight coefficients of objective function
	*T*_*B*_	School bell time
	*T*_*D*_	Departure time from the depot
	*gt*_*j*_	Student gathering time of stop node *j*
	Q	The number of school buses that could be utilized
Sets	*S*	Set of all stop nodes in the network
	*D*	Set of the depot node in the network
	*E*	Set of the school node in the network
	*SD*	Set of all stop nodes and the depot node in the network
	*SE*	Set of all stop nodes and the school node in the network
	*SDE*	Set of all service nodes including the depot, the school, and stop nodes
	*SS*	Sub-set of *S*, |*SS*| represented the number of service nodes in *SS*
	$\Lambda_{ij}^{t}$	Set of candidate paths from service node *i* to service node *j* when departure at time interval *t*
Variables and Parameters	*TC*	Total cost
	*TT*	Total travel time cost
	*WT*	Total waiting time cost
	*WTB*	Total waiting time cost of the school bus
	*WTS*	The waiting time cost of the students for earlier arrival at school
	$x_{ij}^{t}$	If the school bus departs from service node *i* to service node *j* at time interval *t*,$x_{ij}^{t}=1$ otherwise 0
	*t*_*j*_	Time of the school bus arriving at stop node *j*
	*t*_*k*_	Time of the school bus arriving at school
	$\lambda_{ij}^{t}$	Any feasible path from service node *i* to service node *j* when departing at time interval *t* ($\lambda_{ij}^{t}\in \Lambda_{ij}^{t}$)
	$T_{ij}^{t}$	The travel time of path $\lambda_{ij}^{t}$ from service node *i* to service node *j* when departing at time interval *t*
	$\tilde{f}(\lambda_{ij}^{t})$	The worst-case travel time of path $\lambda_{ij}^{t}$
	$\underset{\sim }{f}(\lambda_{ij}^{t})$	The best-case travel time of path $\lambda_{ij}^{t}$
	*Link* (*a*_*k*_, *b*_*k*_)	Links consist in the path $\lambda_{ij}^{t}$ (k=1,2…K)
	$y_{a_{k}b_{k}}^{t}$	If link (*a*_*k*_, *b*_*k*_) was occupied at time interval *t*,$y_{a_{k}b_{k}}^{t}=1$, otherwise 0
	*v*_*iq*_	if service node *i* was visited by vehicle *q*, *v*_*iq* =_ 1; otherwise *v*_*iq*_ = 0

### 3.3 The worst-case and best-case path travel time in STD network

For any path $\lambda_{ij}^{t}$ from service node *i* to service node *j* when departing at time interval *t*, the path travel time in the worst case can be formulated as:

$$\tilde{f}(\lambda_{ij}^{t})=\text{max}\sum_{(a_{k},b_{k})\in \lambda_{ij}^{t}}\sum_{t=0}^{T}C_{a_{k}b_{k}}^{t}\cdot y_{a_{k}b_{k}}^{t}=\text{max}\sum_{(a_{k},b_{k})\in \lambda_{ij}^{t}}\sum_{t=0}^{T}(R_{a_{k}b_{k}}^{t}+\tau_{a_{k}b_{k}}^{t})\cdot y_{a_{k}b_{k}}^{t},\;\tau_{a_{k}b_{k}}^{t}\in \left[0,d_{a_{k}b_{k}}^{t}\right]$$ (3)

At meantime, the path travel time in the best case can be formulated as:

$$\underset{\sim }{f}(\lambda_{ij}^{t})=\text{min}\sum_{(a_{k},b_{k})\in \lambda_{ij}^{t}}\sum_{t=0}^{T}C_{a_{k}b_{k}}^{t}\cdot y_{a_{k}b_{k}}^{t}=\text{min}\sum_{(a_{k},b_{k})\in \lambda_{ij}^{t}}\sum_{t=0}^{T}(R_{a_{k}b_{k}}^{t}+\tau_{a_{k}b_{k}}^{t})\cdot y_{a_{k}b_{k}}^{t},\;\tau_{a_{k}b_{k}}^{t}\in \left[0,d_{a_{k}b_{k}}^{t}\right]$$ (4)

**Proposition:** The above dynamic stochastic function for the worst-case and best-case travel time of path $\lambda_{ij}^{t}$ could be converted into a deterministic time-varying one.

**Fig 1:**
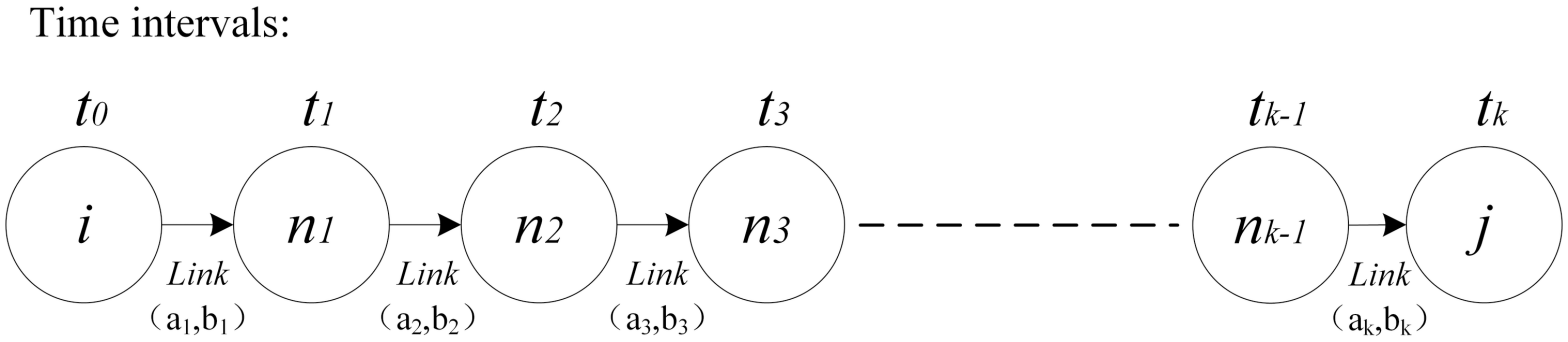
A graphical representation of path $\lambda_{ij}^{t_{\text{0}}}$.

**Proof:** The departure time *T*_*D*_ is set as *t*_0_. We assume that the bus departs from the bus depot node *i* at time interval *t*_0_, and heads to the first stop node *j*, as illustrated in Fig 1. Link $\left(a_{k},b_{k})\in \lambda_{ij}^{t_{0}}(k=1,2\ldots \;K\right)$ represents all the links that consist in the path $\lambda_{ij}^{t_{0}}$ connecting service node *i* with service node *j*. There is no waiting time allowed at any end nodes (*n*_*k*_, *k* = 1,2…*K*-1) of the links (*a*_*k*_, *b*_*k*_) except the stop node *j*. Taking link (*a*_1_, *b*_1_) as an example, the bus departs from node *i* and start to occupy the link (*a*_1_, *b*_1_) at *t*_0_. It is assumed that the bus arrives at the link node *n*_1_ at *t*_1_ which is also the start time to occupy link (*a*_2_, *b*_2_).

According to SCC, for any link $(a_{k},b_{k})\in \lambda_{ij}^{t_{0}}$ at any time interval *t* ≤ *t′*, and any given time *ξ*, the following inequality holds:

$$\text{Pr}\;(C_{a_{k}b_{k}}^{t}+t\leq \xi )\leq \text{Pr}\;(C_{a_{k}b_{k}}^{t^{\prime }}+t^{\prime }\leq \xi )$$ (5)

Thus, it can be rewritten as:

$$\text{Pr}\;(R_{a_{k}b_{k}}^{t}+\tau_{a_{k}b_{k}}^{t}+t\leq \xi )\geq \text{Pr}\;(R_{a_{k}b_{k}}^{t^{\prime }}+\tau_{a_{k}b_{k}}^{t^{\prime }}+t^{\prime }\leq \xi )$$ (6)

**Step1**:

$C_{a_{1}b_{1}}^{t_{0}}$ represents the travel time of link (*a*_1_, *b*_1_), and $R_{a_{1}b_{1}}^{t_{0}}\leq C_{a_{1}b_{1}}^{t_{0}}\leq R_{a_{1}b_{1}}^{t_{0}}+d_{a_{1}b_{1}}^{t_{0}}$. Then, the arrival time of end node *n*_1_ can be formulated as following:

$$t_{\text{1}}=\;C_{a_{1}b_{1}}^{t_{0}}+t_{\text{0}},\;\;R_{a_{1}b_{1}}^{t_{0}}+t_{\text{0}}\leq t_{\text{1}}\leq R_{a_{1}b_{1}}^{t_{0}}+d_{a_{1}b_{1}}^{t_{0}}+t_{\text{0}}$$ (7)

We define $max\;t_{\text{1}}=R_{a_{1}b_{1}}^{t_{0}}+d_{a_{1}b_{1}}^{t_{0}}+t_{\text{0}},\;min\;t_{\text{1}}=R_{a_{1}b_{1}}^{t_{0}}+t_{\text{0}}$. For any departure time *t*_0_, the inequality *mint*_1_ ≤ *t*_1_ ≤ *maxt*_1_ always holds.

**Step 2**:

As it is defined, there is no waiting time at the previous node before moving forward to the next, so we assume that the bus departs from link node *n*_1_ at time *t*_1_ and arrives at the link node *n*_2_ at time *t*_2_ through link (*a*_2_, *b*_2_). Then, the arrival time can be represented as $t_{\text{2}}=C_{a_{\text{2}}b_{\text{2}}}^{t_{\text{1}}}+t_{\text{1}}$.

**Step 2(a):** Since *t*_1_ ≤ *maxt*_1_, the following equality can be obtained:

$$\text{Pr}\;(C_{a_{\text{2}}b_{\text{2}}}^{t_{\text{1}}}+t_{\text{1}}\leq \xi )\geq \text{Pr}\;(C_{a_{\text{2}}b_{\text{2}}}^{max\;t_{\text{1}}}+\text{max}t_{\text{1}}\leq \xi )$$ (8)

We define $\xi =\;max(C_{a_{\text{2}}b_{\text{2}}}^{max\;t_{\text{1}}}+\text{max}t_{\text{1}})$, then the inequality is equivalent to:

$$Pr\;\left(C_{a_{\text{2}}b_{\text{2}}}^{t_{\text{1}}}+t_{\text{1}}\leq max(C_{a_{\text{2}}b_{\text{2}}}^{max\;t_{\text{1}}}+\text{max}t_{\text{1}})\right)\geq Pr\left(C_{a_{\text{2}}b_{\text{2}}}^{max\;t_{\text{1}}}+\text{max}t_{\text{1}}\leq max(C_{a_{\text{2}}b_{\text{2}}}^{max\;t_{\text{1}}}+\text{max}t_{\text{1}})\right)$$ (9)

It could be easily found that the probability of the right-side formula is 100%, so we can have:

$$\text{Pr}\left(C_{a_{\text{2}}b_{\text{2}}}^{t_{\text{1}}}+t_{\text{1}}\leq max\left(C_{a_{\text{2}}b_{\text{2}}}^{max\;t_{\text{1}}}+\text{max}t_{\text{1}}\right)\right)\geq \text{1}$$ (10)

Then we can determine that if the above-mentioned inequality always holds, it must have the following relation always holds:

$$t_{\text{2}}\leq max\left(C_{a_{\text{2}}b_{\text{2}}}^{max\;t_{\text{1}}}+\text{max}t_{\text{1}}\right),\;t_{\text{2}}=C_{a_{\text{2}}b_{\text{2}}}^{t_{\text{1}}}+t_{\text{1}}$$ (11)

That is,

$$t_{\text{2}}\leq max\left(C_{a_{\text{2}}b_{\text{2}}}^{max\;t_{\text{1}}}+\text{max}t_{\text{1}}\right)=\text{max}(R_{a_{\text{2}}b_{\text{2}}}^{max\;t_{1}}+\tau_{a_{\text{2}}b_{\text{2}}}^{max\;t_{1}}+\text{max}t_{\text{1}})=R_{a_{\text{2}}b_{\text{2}}}^{max\;t_{1}}+d_{a_{\text{2}}b_{\text{2}}}^{max\;t_{1}}+R_{a_{1}b_{1}}^{t_{0}}+d_{a_{1}b_{1}}^{t_{0}}+t_{\text{0}}$$ (12)

**Step 2(b):** Besides, since *t*_1_ ≥ min *t*_1_, then the following equality should hold:

$$\text{Pr}\;(C_{a_{\text{2}}b_{\text{2}}}^{\text{min}t_{\text{1}}}+\text{min}t_{\text{1}}\leq \xi )\geq \text{Pr}\;(C_{a_{\text{2}}b_{\text{2}}}^{t_{\text{1}}}+t_{\text{1}}\leq \xi )$$ (13)

We define $\xi <\;\text{min}(C_{a_{\text{2}}b_{\text{2}}}^{\text{min}t_{\text{1}}}+\text{min}t_{\text{1}})$, then the inequality is equivalent to:

$$\text{Pr}\;(C_{a_{\text{2}}b_{\text{2}}}^{\text{min}t_{\text{1}}}+\text{min}t_{\text{1}}<\text{min}(C_{a_{\text{2}}b_{\text{2}}}^{\text{min}t_{\text{1}}}+\text{min}t_{\text{1}}))\geq \text{Pr}\;(C_{a_{\text{2}}b_{\text{2}}}^{t_{\text{1}}}+t_{\text{1}}<\text{min}(C_{a_{\text{2}}b_{\text{2}}}^{\text{min}t_{\text{1}}}+\text{min}t_{\text{1}}))$$ (14)

It could be easily found that the probability of the left-side formula is 0%, so we can have:

$$\text{Pr}\;(C_{a_{\text{2}}b_{\text{2}}}^{t_{\text{1}}}+t_{\text{1}}<\text{min}(C_{a_{\text{2}}b_{\text{2}}}^{\text{min}t_{\text{1}}}+\text{min}t_{\text{1}}))\text{=0}$$ (15)

Then we can determine that if the above-mentioned inequality always holds, it must have the following relation always holds:

$$t_{\text{2}}\geq \text{min}\left(C_{a_{\text{2}}b_{\text{2}}}^{\text{min}t_{\text{1}}}+\text{min}t_{\text{1}}\right),\;t_{\text{2}}=C_{a_{\text{2}}b_{\text{2}}}^{t_{\text{1}}}+t_{\text{1}}$$ (16)

That is,

$$t_{2}\geq \text{min}\left(C_{a_{2}b_{2}}^{\text{min}t_{1}}+\text{min}t_{1}\right)=\text{min}(R_{a_{2}b_{2}}^{\text{min}t_{1}}+\tau_{a_{2}b_{2}}^{\text{min}t_{1}}+\text{min}t_{1})=R_{a_{2}b_{2}}^{\text{min}t_{1}}+R_{a_{1}b_{1}}^{t_{0}}+t_{0}$$ (17)

We define $max\;t_{\text{2}}=R_{a_{\text{2}}b_{\text{2}}}^{max\;t_{1}}+d_{a_{\text{2}}b_{\text{2}}}^{max\;t_{1}}+R_{a_{1}b_{1}}^{t_{0}}+d_{a_{1}b_{1}}^{t_{0}}+t_{\text{0}},\;min\;t_{\text{2}}=R_{a_{2}b_{2}}^{\text{min}t_{1}}+R_{a_{1}b_{1}}^{t_{0}}+t_{0}$. Since *mint*_1_ and *maxt*_1_ can also be written as the function of departure time *t*_0_. Thus, for any time *t*_0_, the inequality *mint*_2_ ≤ *t*_2_ ≤ *maxt*_2_ always holds.

Then Step 3, Step 4 until Step m-1, the school bus arrives at the first stop node *j*.

**Step m-1**:

Assuming that the school bus arrives at the destination node *j* at time period *t*_*k*_, then we can obtain the following two recursion formulas:

Recursion formula 1:

$$\begin{array}{l} t_{k}={\text{C}}_{a_{k}b_{k}}^{t_{k-1}}+t_{k-1}\leq \text{max}\;{\text{C}}_{a_{k}b_{k}}^{\text{max}\;t_{k-1}}+\text{max}\;{\text{t}}_{k-1}={\text{R}}_{a_{k}b_{k}}^{\text{max}\;t_{k-1}}+{\text{d}}_{a_{k}b_{k}}^{\text{max}t_{k-1}}+\\ \cdots \cdots +R_{a_{\text{2}}b_{\text{2}}}^{max\;t_{1}}+d_{a_{\text{2}}b_{\text{2}}}^{max\;t_{1}}+R_{a_{1}b_{1}}^{t_{0}}+d_{a_{1}b_{1}}^{t_{0}}+t_{\text{0}} \end{array}$$ (18)

Recursion formula 2:

$$\;t_{k}{=\;\text{C}}_{a_{k}b_{k}}^{{\text{t}}_{k-1}}+t_{k-1}\geq \text{min}C_{a_{k}b_{k}}^{\text{min}t_{k-1}}+\text{min}{\text{t}}_{k-1}={\text{R}}_{a_{k}b_{k}}^{\text{min}t_{k-1}}+\cdots \cdots +R_{a_{2}b_{2}}^{\text{min}t_{1}}+R_{a_{1}b_{1}}^{t_{0}}+t_{0}$$ (19)

It can be rewritten as following:

$$T_{ij}^{t_{0}}=t_{k}-t_{0}$$ (20)

$$T_{ij}^{t_{0}}\leq {\text{R}}_{a_{k}b_{k}}^{\text{max}t_{k-1}}+{\text{d}}_{a_{k}b_{k}}^{\text{max}t_{k-1}}+\cdots +R_{a_{\text{2}}b_{\text{2}}}^{max\;t_{1}}+d_{a_{\text{2}}b_{\text{2}}}^{max\;t_{1}}+R_{a_{1}b_{1}}^{t_{0}}+d_{a_{1}b_{1}}^{t_{0}}=\overset{K}{\underset{k=1}{\sum }}\overset{T}{\underset{t=t_{0}}{\sum }}\left(R_{a_{k}b_{k}}^{t}+d_{a_{k}b_{k}}^{t}\right)•y_{a_{k}b_{k}}^{t}$$ (21)

$$\;T_{ij}^{t_{0}}\geq {\text{R}}_{a_{k}b_{k}}^{\text{min}t_{k-1}}+\cdots \cdots +R_{a_{2}b_{2}}^{\text{min}t_{1}}+R_{a_{1}b_{1}}^{t_{0}}+t_{0}=\overset{K}{\underset{k=1}{\sum }}\overset{T}{\underset{t=t_{0}}{\sum }}R_{a_{k}b_{k}}^{t}•y_{a_{k}b_{k}}^{t}$$ (22)

$(a_{k},b_{k})\in \lambda_{ij}^{t_{0}}$, and $y_{a_{k}b_{k}}^{t}=1$ when the school bus starts to occupy links (*a*_*k*_, *b*_*k*_) at time interval *t*, otherwise 0.

For any time *t*_0_, $\;\sum_{k=1}^{K}\sum_{t=t_{0}}^{T}R_{a_{k}b_{k}}^{t}•y_{a_{k}b_{k}}^{t}\leq T_{ij}^{t_{0}}\leq \sum_{k=1}^{K}\sum_{t=t_{0}}^{T}\left(R_{a_{k}b_{k}}^{t}+d_{a_{k}b_{k}}^{t}\right)•y_{a_{k}b_{k}}^{t},\;(a_{k},b_{k})\in \lambda_{ij}^{t_{0}}$ always holds. Thus, the worst-case and best-case travel time of path $\lambda_{ij}^{t_{\text{0}}}$ can be obtained:

$$\tilde{f}(\lambda_{ij}^{t_{0}})=\;Max\;T_{ij}^{t_{0}}=\overset{K}{\underset{k=1}{\sum }}\overset{T}{\underset{t=t_{0}}{\sum }}\left(R_{a_{k}b_{k}}^{t}+d_{a_{k}b_{k}}^{t}\right)•y_{a_{k}b_{k}}^{t}$$ (23)

$$\;\underset{\sim }{f}(\lambda_{ij}^{t_{0}})=Min\;T_{ij}^{t_{0}}=\overset{K}{\underset{k=1}{\sum }}\overset{T}{\underset{t=t_{0}}{\sum }}R_{a_{k}b_{k}}^{t}•y_{a_{k}b_{k}}^{t}$$ (24)

$(a_{k},b_{k})\in \lambda_{ij}^{t_{0}}$, and $y_{a_{k}b_{k}}^{t}=1$ when the school bus starts to occupy links (*a*_*k*_, *b*_*k*_) at time interval *t*, otherwise 0.

In Eqs (23) and (24), $R_{a_{k}b_{k}}^{t}$ and $R_{a_{k}b_{k}}^{t}\text{+}d_{a_{k}b_{k}}^{t}$ are respectively the lower and upper bound of travel time of link (*a*_*k*_, *b*_*k*_) at time interval *t*, which are dynamic but deterministic at each time interval. Thus, it has been proved that the dynamic stochastic function for the worst-case and best-case travel time of path $\lambda_{ij}^{t}$ could be converted into a deterministic time-varying one.

### 3.4 Robust Optimal Schedule Time model

**Fig 2:**
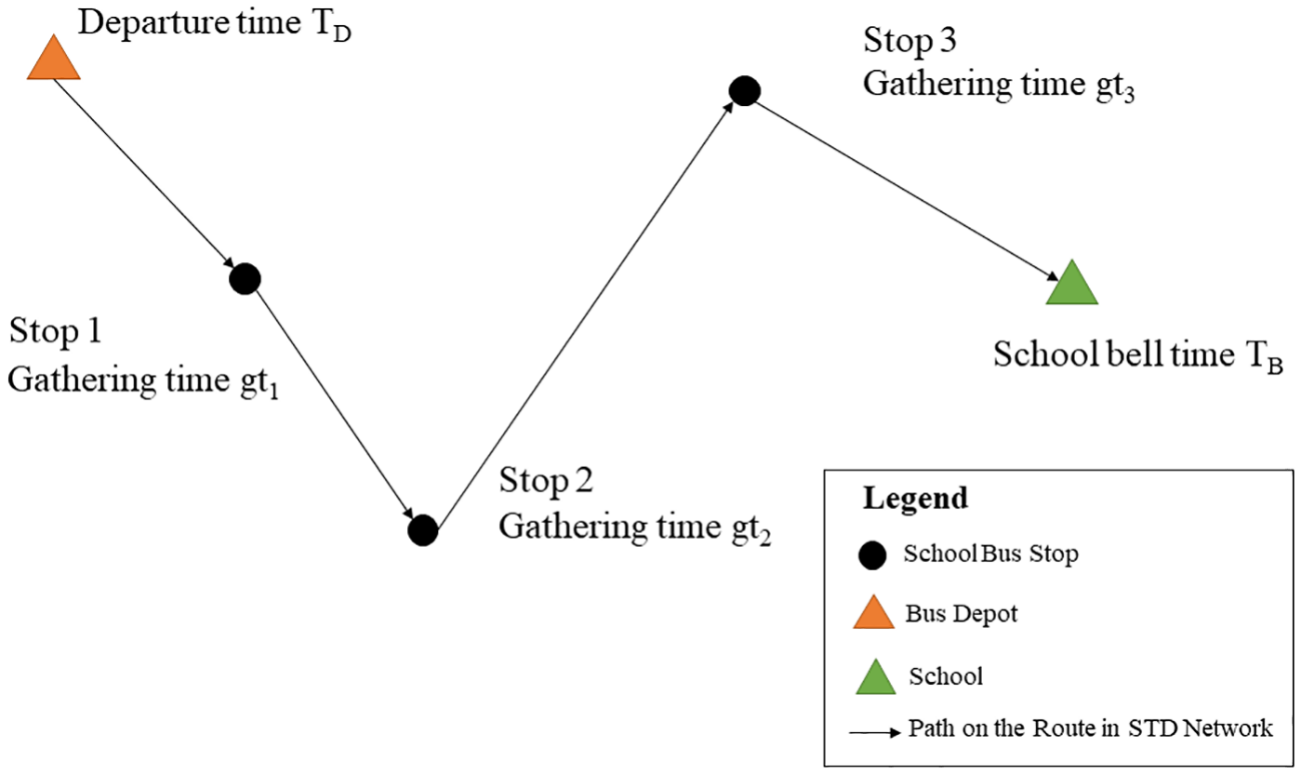
An example of the school bus route.

This paper addressed the SBRP based on a single-school configuration. In addition, it was also assumed that the students were picked up at their homes according to the features of school bus systems in China. Therefore, the location of each student’s house could be regarded as the bus stop, and the school bus left the depot in the morning at a fixed time, then picked up the students one by one. As shown in Fig 2, it provided an example of the school bus route. The gathering time at each bus stop represented the appointed departure time, before which the school bus should have already arrived at the student’s house and waited for the student to get on board. At meanwhile, it also indicated that the school bus should arrive at the school before the school bell time, otherwise the students were late for school. Thus, it was assumed that the gathering time and the school bell time were defined as hard time windows in this paper. It implied that any delays were not allowed when picked up students and delivered them to the school. Although being late for school was not permitted in the current study, whereas, arriving too early meant a waste of time, so arriving around the school bell time in the morning might be the best desire. Similarly, it was also more desired to reduce the waiting time of school bus as much as possible when arriving at each student’s house. Thus, the cost of earlier arrival should be considered in this context, and it was defined as “earlier schedule delay”.

**Fig 3:**
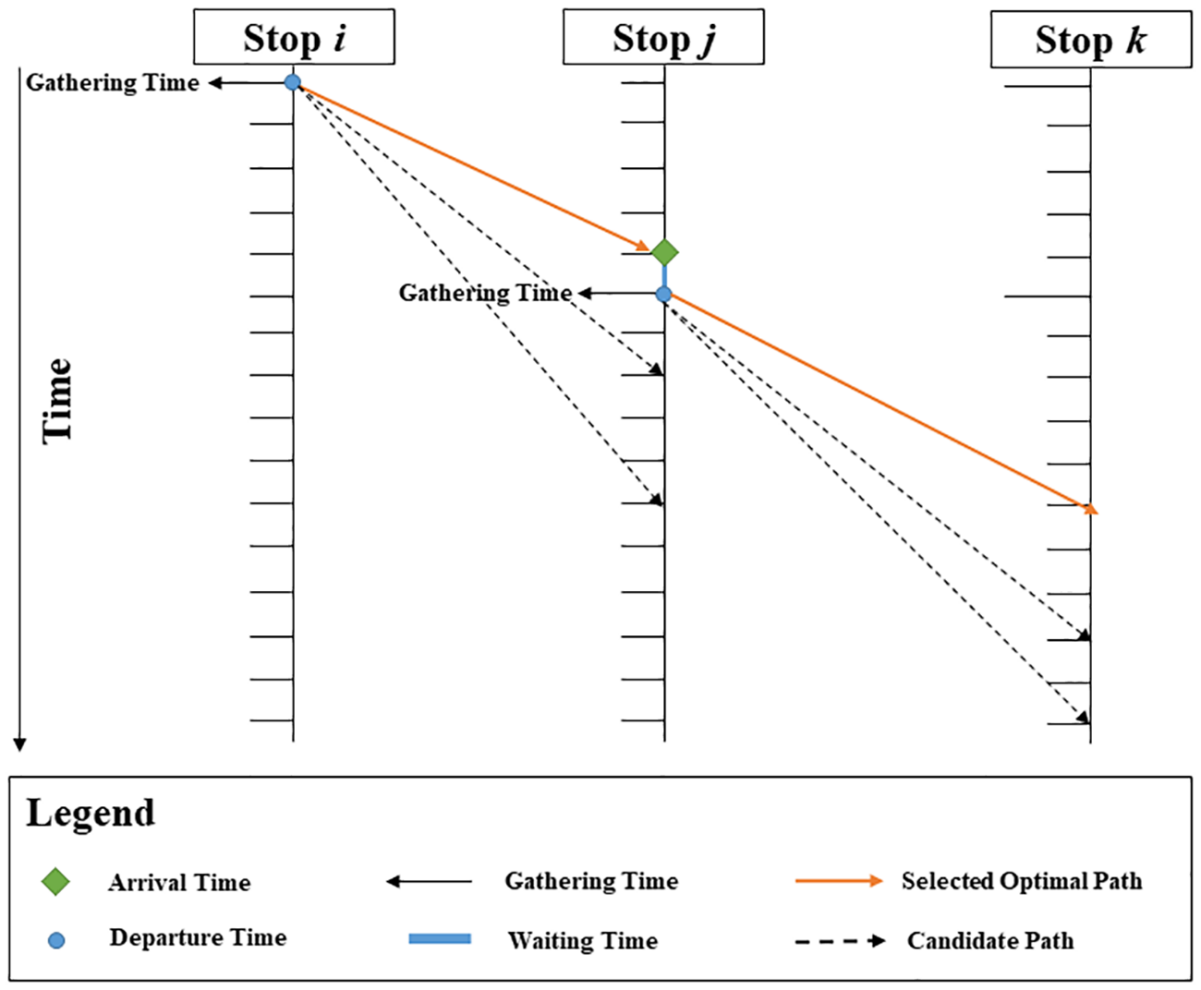
An illustration of SBRP in STD networks.

In this paper, the cost of earlier schedule delay combined with the disutility of travel times were referred as the evaluation index, in which the former one is defined as the cost due to the earlier arrival. In this context, the fixed cost of the already purchased bus fleet was not taken into account, including the purchased fees, drivers’ salary, and parking fees. This part of cost had already been paid, and had no effect on the new bus route generation. However, the number of school buses that could be utilized was limited, and no more new buses would be added. The incremental cost of school bus operation was based on per unit time travelled, so the cost of total travel times increased along with all utilized buses traveling, indirectly considering the effect of the number of buses actually used (N), without regard to their fixed cost. Moreover, it was also assumed that the school buses were homogenous with unlimited capacity. The main goal of this study was to generate an efficient route for the school bus. However, the travel times here were treated as random dynamic variables to account for the nature of real transportation network. The uncertainties of travel times led to the changes in optimality criteria of paths selecting. Thus, in addition to determining the optimal sequence of service nodes in the route, it was also necessary to find the optimal path connecting the current service node with the next one, as shown in Fig 3.

To deal with the uncertainties, the Min-Max approach derived from robust optimization was employed in this paper to evaluate the performance. Since the link travel times were attributed to both stochastic and time-dependent, the arrival time at each stop and at the final destination was uncertain. Therefore, the earlier schedule delay as well as the total cost was also varying within a range. Through the adopted robust approach, it could seek to find a more cost reliable route by minimizing the upper bound of the total cost of all the feasible solutions. Thus, a routing choice with the most robust schedule time was viewed as the optimal one. The Robust Optimal Schedule Time Model (ROSTM) for school bus routing was formulated as follows, which minimized the total cost in the worst case of candidate solutions.

**Objective function**:

Min(Maxz) (25)

z=TC (26)

**Expressions of the objective function**:

$$WTB=\alpha_{\text{2}}•\sum_{j\in S}\left(gt_{j}-t_{j}\right)$$ (27)

$$WTS=\alpha_{\text{2}}•(T_{B}-t_{k})$$ (28)

$$t_{j}=\{\begin{array}{c} {\text{gt}}_{j-1}+T_{(j-1)j}^{{\text{gt}}_{j-1}},\;\;\;\;j\in S,j\neq the\;first\;stop\;node\;\;\;\;\;\;\\ T_{D}+T_{ij}^{T_{D}},\;\;i=depot\;node,\;j=the\;first\;stop\;node \end{array}$$ (29)

$$t_{k}={\text{gt}}_{l}+T_{lk}^{{\text{gt}}_{l}},\;k=\;school\;node,\;l=the\;last\;stop\;node$$ (30)

$$WT=WTB+WTS=\alpha_{\text{2}}•\sum_{j\in S}\left(gt_{j}-t_{j}\right)\text{+}\alpha_{\text{2}}•(T_{B}-t_{k})=\alpha_{\text{2}}•\left[T_{B}-T_{D}-\sum_{i\in SD}\sum_{\begin{array}{l} j\in SD\\ j\neq i \end{array}}\sum_{t=T_{D}}^{T}\left(T_{ij}^{t}\cdot x_{ij}^{t}\right)\right]$$ (31)

$$TT=\alpha_{1}•\sum_{i\in SD}\sum_{\begin{array}{l} j\in SD\\ j\neq i \end{array}}\sum_{t=T_{D}}^{T}\left(T_{ij}^{t}\cdot x_{ij}^{t}\right)$$ (32)

$$\begin{array}{l} TC=TT+WT=\alpha_{1}•\sum_{i\in SD}\sum_{\begin{array}{l} j\in SD\\ j\neq i \end{array}}\sum_{t=T_{D}}^{T}\left(T_{ij}^{t}\cdot x_{ij}^{t}\right)+\alpha_{\text{2}}•\left[T_{B}-T_{D}-\sum_{i\in SD}\sum_{\begin{array}{l} j\in SD\\ j\neq i \end{array}}\sum_{t=T_{D}}^{T}\left(T_{ij}^{t}\cdot x_{ij}^{t}\right)\right]\\ \;\;\;=(\alpha_{1}-\alpha_{2})•\sum_{i\in SD}\sum_{\begin{array}{l} j\in SD\\ j\neq i \end{array}}\sum_{t=T_{D}}^{T}\left(T_{ij}^{t}\cdot x_{ij}^{t}\right)+\alpha_{\text{2}}•(T_{B}-T_{D}) \end{array}$$ (33)

$$\begin{array}{l} Min\;(Max\;TC)=Min\;[\;\;\;(\alpha_{1}-\alpha_{2})•Max\sum_{i\in SD}\sum_{\begin{array}{l} j\in SD\\ j\neq i \end{array}}\sum_{t=T_{D}}^{T}\left(T_{ij}^{t}\cdot x_{ij}^{t}\right)+\alpha_{\text{2}}•(T_{B}-T_{D})]\\ \;\;\;\;=Min\;\;[(\alpha_{1}-\alpha_{2})•\sum_{i\in SD}\sum_{\begin{array}{l} j\in SD\\ j\neq i \end{array}}\overset{K}{\underset{k=1}{\sum }}\overset{T}{\underset{t=T_{D}}{\sum }}\left(R_{a_{k}b_{k}}^{t}+d_{a_{k}b_{k}}^{t}\right)•y_{a_{k}b_{k}}^{t}•x_{ij}^{t}]+\alpha_{\text{2}}•(T_{B}-T_{D}))\;\;(a_{k},b_{k})\in \lambda_{ij}^{T_{D}} \end{array}$$ (34)

**Subject to**:

$$\alpha_{1}>\alpha_{2}$$ (35)

$$T_{B}>t_{k}$$ (36)

$$gt_{j}\geq t_{j}$$ (37)

$$\sum_{\begin{array}{l} i\in SD\\ j\neq i \end{array}}\sum_{t=0}^{T}x_{ij}^{t}=1,\;j\in SE$$ (38)

∑j∈SDi≠j∑t=0Txijt=1,i∈SD (39)

∑q=1Qviq=1,i∈S (40)

∑q=1Qq(viq−vjq)≤B(1−∑t=0Txijt),i∈SD,j∈SE,i≠j (41)

∑q=1Qq(viq−vjq)≥B(∑t=0Txijt−1),i∈SD,j∈SE,j≠i (42)

∑i,j∈SSi≠j∑t=0Txijt≤|SS|−1,SS∈D (43)

The objective function (Eq 25) minimized the total cost in the worst case to obtain a cost reliable route. The total cost (Eq 33) included the cost of total travel times (Eq 32) and the cost of earlier schedule delays (Eq 31). The latter one consisted of the waiting time cost of the school bus at each stop (Eq 27) and the earlier arrival cost of students at school (Eq 28). *α*_1_ and *α*_2_ were weight coefficients. In order to avoid the intentional detours through which the waiting time could be reduced, *α*_1_ should be assigned a larger value (Eq 35). Eq (34) indicated that the objective function could be converted into solving a shortest path (SP) problem in a deterministic time-dependent network, since $R_{a_{k}b_{k}}^{t}\text{+}d_{a_{k}b_{k}}^{t}$ was only dynamic. Constraint (Eqs 36 and 37) stated that no delays of school buses were allowed at each stop or the final destination. Constraint (Eqs 38 and 39) stated that each service node could be visited only once. Constraint (Eqs 40–42) stated that the service node in each route could only be served by one school bus. Constraint (Eq 43) was added for route continuity.

In the above ROST model, the departure time from the depot, the gathering time at each stop, and the school bell time were all fixed. It was assumed that the dynamic and stochastic travel time could be derived from priori information of traffic conditions provided by Advanced Traveler Information System (ATIS). Furthermore, according to Eq (34), the efficient route could be computed by solving a variant of a conventional VRP in a deterministic dynamic network, in which the edge weight had been converted into the dynamic function of the upper bound value of link travel times. Fortunately, the computational complexity of the converted model was much lower than the original STD one, which provided the potential of applying to real-life transportation networks [25, 26].

## 4 Computational instances and analysis

### 4.1 Computational instances

**Fig 4:**
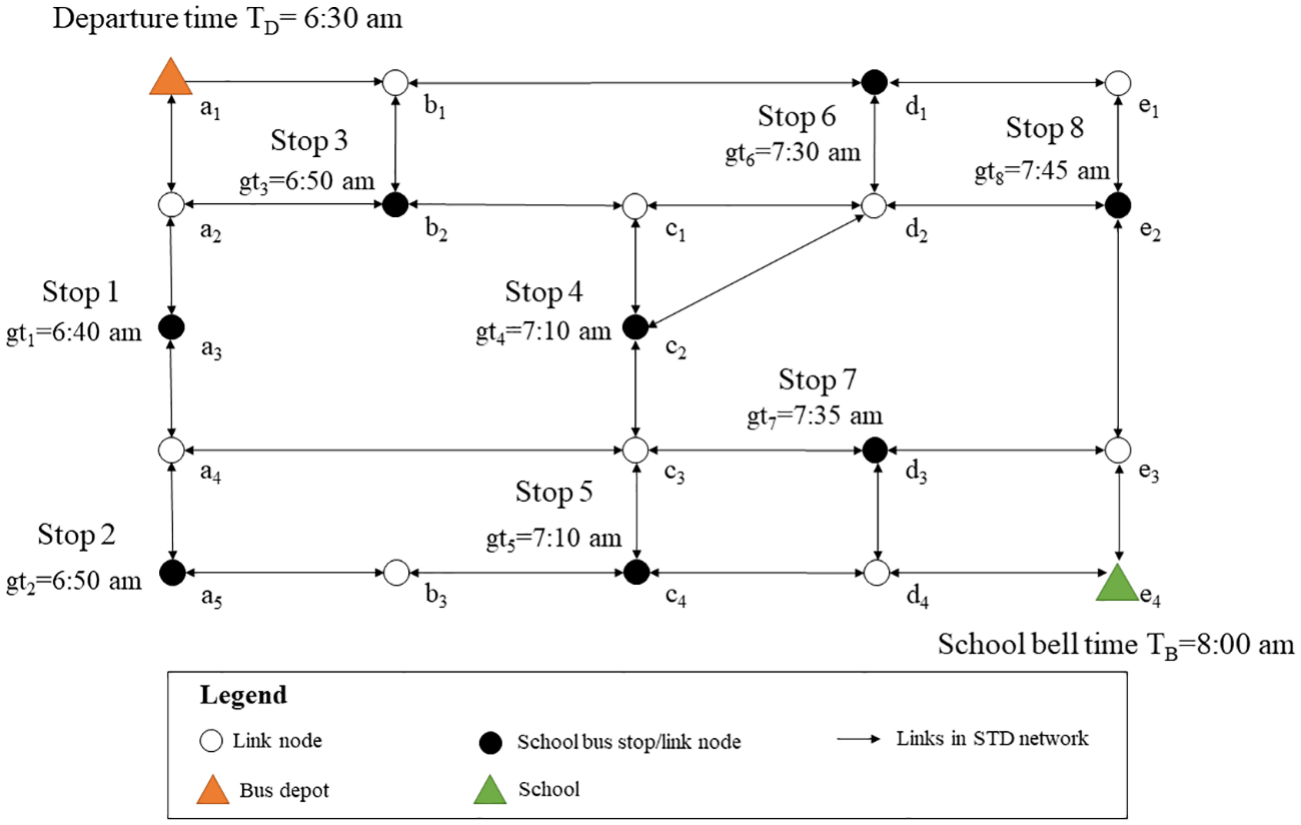
The computational instance in the test.

In this paper, the transportation network was extended, in which the link travel times were attributed to both dynamic and stochastic. However, the school bus route generation problem in STD networks could be converted and regarded as a variant of time-dependent VRP (TDVRP). Nevertheless, it was also a NP-hard problem although the computational complexity had been decreased compared with the original one. To better evaluate the performance of ROST model by finding the exact optimal solution, a small-scale computational instance was designed in the test, as shown in Fig 4. In addition, in order to design and complete the instances, the number of school bus which could be used in operation was up to 2, and *α*_1_, *α*_2_ were respectively set as 1 and 0.5.

The time-dependent function of link travel times and their stochastic fluctuation characteristics were also simulated to represent the nature of transportation networks in the morning peak-hours. The morning peak (6:30 am-8:00 am) was segmented into 3 time intervals: T1 = [6:30 am-7:00 am); T2 = [7:00 am-7:30 am); T3 = [7:30 am-8:00 am], which respectively represented the start stage, the medium stage and the end stage. Since the travel times and the level of fluctuation in each stage was varying, a time-dependent piecewise function should be established, associated with different levels of fluctuations. As shown in Table 3, the traffic status presented in table cells was the lower bound (LB) of link travel times corresponding to each time interval. It was also assumed that the traffic status on the two directions of the same link was consistent, e.g. link (*a*_1_, *a*_2_) and link (*a*_2_, *a*_1_) shared the same travel time and its fluctuation characteristics at each time interval.

**Table 3:** The lower bound of link travel times at each time interval.

Link	LB at T1, T2, T3	Link	LB at T1, T2, T3
(*a*_1_, *a*_2_)	3 mins, 5 mins, 7 mins	(*a*_1_, *b*_1_)	10 mins, 12 mins, 14 mins
(*a*_2_, *a*_3_)	4 mins, 6 mins, 8 mins	(*a*_2_, *b*_2_)	10 mins, 12 mins, 14 mins
(*a*_3_, *a*_4_)	5 mins, 7 mins, 9 mins	(*a*_4_, *a*_5_)	2 mins, 4 mins, 6 mins
(*a*_4_, *c*_3_)	20 mins, 22 mins, 24 mins	(*a*_5_, *b*_3_)	10 mins, 12 mins, 14 mins
(*b*_1_, *b*_2_)	5 mins, 7 mins, 9 mins	(*b*_1_, *d*_1_)	15 mins, 17 mins, 19 mins
(*b*_2_, *c*_1_)	10 mins, 12 mins, 14 mins	(*b*_3_, *c*_4_)	3 mins, 5 mins,7 mins
(*c*_1_, *c*_2_)	3 mins, 5 mins, 7 mins	(*c*_1_, *d*_2_)	3 mins, 5 mins, 7 mins
(*c*_2_, *c*_3_)	13 mins, 15 mins, 17 mins	(*c*_2_, *d*_2_)	8 mins, 10 mins, 12 mins
(*c*_3_, *c*_4_)	8 mins, 10 mins, 12 mins	(*c*_3_, *d*_3_)	8 mins, 10 mins, 12 mins
(*c*_4_, *d*_4_)	15 mins, 17 mins, 17 mins	(*d*_1_, *d*_2_)	3 mins, 5 mins,7 mins
(*d*_1_, *e*_1_)	3 mins, 3 mins, 5 mins	(*d*_2_, *e*_2_)	8 mins, 10 mins, 10 mins
(*d*_3_, *d*_4_)	2 mins, 2 mins, 8 mins	(*d*_3_, *e*_3_)	3 mins, 3 mins, 5 mins
(*d*_4_, *e*_4_)	8 mins, 10 mins, 12 mins	(*e*_1_, *e*_2_)	3 mins, 3 mins, 5 mins
(*e*_2_, *e*_3_)	3 mins, 3 mins, 5 mins	(*e*_3_, *e*_4_)	3 mins, 3 mins, 5 mins

In order to describe the random fluctuation of traffic status, three levels of travel time fluctuation were defined in Table 4, respectively corresponding to each stage in the morning peak-hours. Then, the time-dependent piecewise function of link travel times could be simulated by taking account of the lower bound of travel times and the fluctuation range.

**Table 4:** Fluctuations of travel time.

Fluctuation level	Fluctuation range
Low (Start stage at T1)	Up to 10%
Medium (Medium stage at T2)	Up to 20%
High (End stage at T3)	Up to 30%

### 4.2 The programming solver and test environment

In order to find the exact optimal solution of ROST model in the current computational instance, a mathematical programming solver was adopted, namely CPLEX. It could provide powerful algorithms to produce precise and logical solutions, and also enable decision optimization for the improvement of efficiency. The computation and test environment in this paper was as follows:

- CPU: Intel Core i7-7820HQ (8 cores), 2.90GHz;
- RAM: 16GB;
- Operation system: Windows 10 Home Basic Version (64bit);
- Programming solver: IBM ILOG CPLEX Optimization Studio.

### 4.3 Solutions and discussions

**Fig 5:**
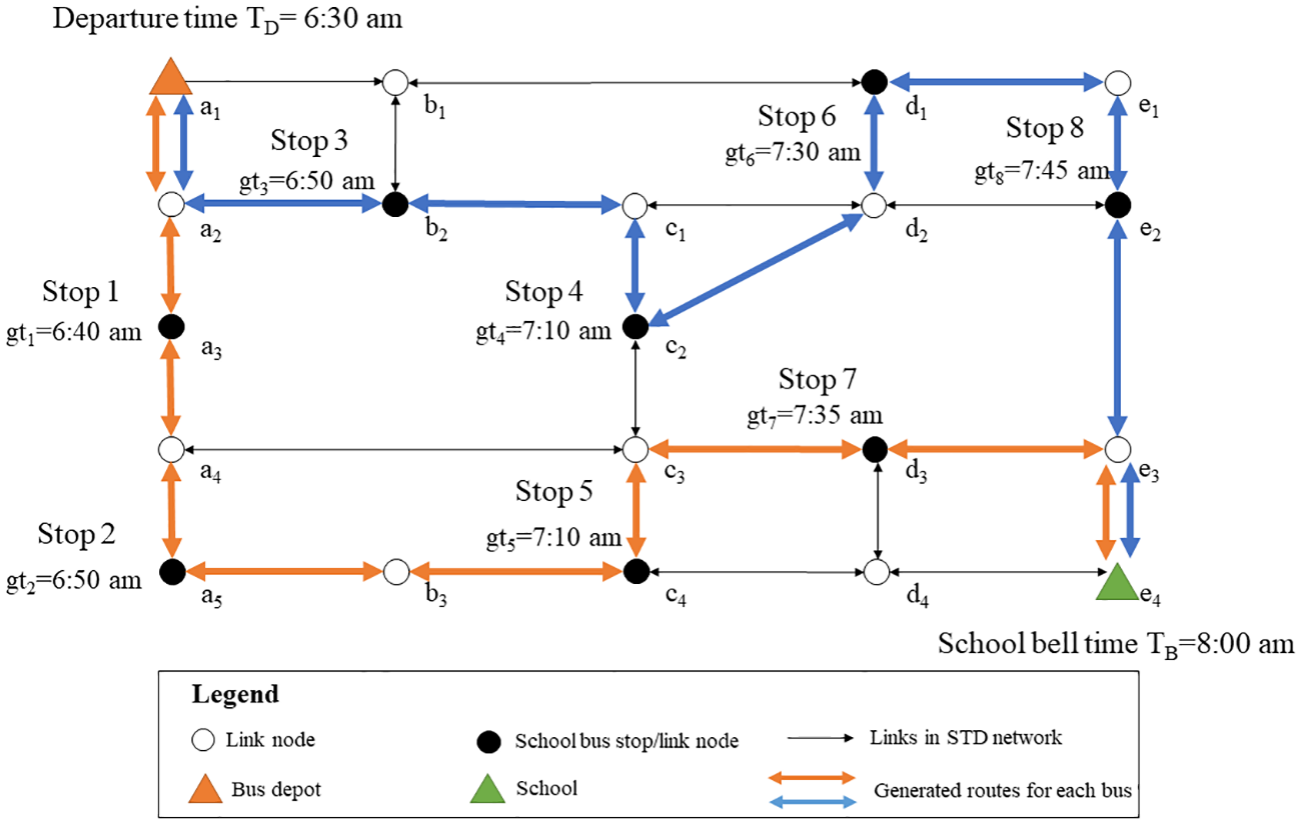
The generated routes for utilized school bus.

The problem based on the designed computational instance was solved by the use of CPLEX. Finally, two school buses were utilized to pick up students. The solutions, namely the optimal routes for the operation of school buses were illustrated in Fig 5.

Specifically, the generated route for the first school bus was denoted in orange, as shown in Fig 5. The bus departed from the depot at 6:30 am, and respectively picked up students at Stop 1, Stop 2, Stop 5, and Stop 7 in sequence. At the same time, the generated route for the second school bus (the blue one in Fig 5) respectively visited the remaining stops one by one. Since the travel times were uncertain, the arrival time of the first school bus at destination was also a time range, which could be calculated based on the generated route as [7:45 am, 7:48 am]. Similarly, the arrival time at school of the second bus took range from 7:55 am to 7:58 am. The arrival times of the school buses at each stop could also be computed in the same way. The results indicated that both the two school bus could arrive before the school bell time, and also satisfy the gathering time window at each stop either, due to the constraints in the model.

However, when the links and nodes of the generated school bus routes were examined, some findings could be derived from the test to reflect the importance of considering the uncertain and dynamic nature of transportation networks. Taking the route (*a*_1_-*a*_2_-*a*_3_-*a*_4_-*a*_5_-*b*_3_-*c*_4_-*c*_3_-*d*_3_-*e*_3_-*e*_4_) of the first school bus for example, when the school bus left Stop 5 and headed to Stop 7, it finally selected the path (*c*_4_-*c*_3_-*d*_3_) instead of the path (*c*_4_-*d*_4_-*d*_3_). The possible arrival time could be calculated for each link node of the two candidate paths connecting Stop 5 with Stop 7. Since the school bus was known to depart from Stop 5 at 7:10 am, the travel times of link (*c*_4_-*c*_3_) and link (*c*_4_-*d*_4_) at T2 could be respectively searched from Tables 3 and 4. The possible arrival time at link node *c*_3_ took range from 7:20 am to 7:22 am, while the school bus may arrived at link node *d*_4_ in the time range of [7:27 am, nearly 7:31 am]. Nevertheless, the travel times of the next adjacent link (*c*_3_-*d*_3_) in the selected path could still take value at T2, whereas link (*d*_4_-*d*_3_) were possible to take the value of travel times at T2 as well as T3 due to the uncertainty of arrival time at link node *d*_4_. If the school bus arrived at link node *d*_4_ before 7:30 am, then it could at least got to Stop 7 before 7:33 am, which implied that the path (*c*_4_-*d*_4_-*d*_3_) had the possibility to be better than the current selected one (The arrival time at Stop 7 in the selected path ranged from 7:30 am to 7:36 am). However, when the first school bus unfortunately encountered a larger fluctuation of travel times on the link (*c*_4_-*d*_4_) and arrived at link node *d*_4_ after 7:30 am, it would have a risk of being late for the appointed time at Stop 7. Thus, although it would probably not only reduced the waiting time at Stop 7 but also led to a total cost saving, the counterpart of path (*c*_4_-*c*_3_-*d*_3_) should be declined to gain a more time-reliable one.

To compare the results with the model which only considered a dynamic deterministic transportation network, the travel times of link (*c*_4_-*c*_3_) and link (*c*_4_-*d*_4_) at T2 were respectively assumed as a fixed value of 10 minutes and 17minutes, in the same scenario analysis described above. Thus, the arrival time of node *c*_3_ and *d*_4_ could be calculated as 7:20 am and 7:27 am. Furtherly, the time cost of link (*c*_3_-*d*_3_) and link (*d*_4_-*d*_3_) were respectively assumed as 10 minutes and 2 minutes at T2. Obviously, path (*c*_4_-*d*_4_-*d*_3_) was better than the current selected one due to a decrease in the cost of traveling times. However, in reality, the travel times were attributed to both dynamic and stochastic. It had proved in above that choosing path (*c*_4_-*d*_4_-*d*_3_) would have a risk of being late for the appointed time at Stop 7 in a STD road circumstance which was in line with practical situation. Thus, the case analysis reflected that it was necessary to capture the uncertainties and fluctuations of travel times in such a stochastic and time-dependent network, since the reliability of arrival times were also highly concerned by commuters, in addition to the total cost.

## 5 Conclusion

This paper addressed SBRP based on a single-school configuration. Taking the current situation of school bus systems in China into account, several common assumptions were adopted such as a homogeneous bus fleet, picking up students at their homes, and fixed school bell time. However, different from the existing work in the literature, the stochastic and time-varying characteristics of travel times in the transportation network were concerned in this study. Due to the fluctuation and uncertainty of travel times, the cost of candidate paths connecting the current service node with the next one were varying. Therefore, the optimal path selecting problem in STD networks was regarded as a sub-problem of SBRP. Meanwhile, the arrival time at each stop node including the destination, was also uncertain within a range. Since the reliability of travel times was highly concerned by such time-rigid commuters, a hard time window constraint was employed in the model, and the cost of earlier schedule delays combined with the disutility of travel times were proposed as the evaluation index. In order to generate a most cost-reliable route for school buses, a Min-Max approach derived from RO was applied to STD instances, and then the ROST model was formulated which minimized the upper bound of the total cost of all the feasible solutions. It also proved that the objective function in the ROST model could be simplified for a reduction of computation complexity, and converted into solving a variant of conventional TDVRP which was based on the worst-case link travel times. Then, a small-scale computational instance was designed and analyzed to verify the validity of the proposed model and evaluate its performance. A mathematical programming solver, namely CPLEX, was used to find the exact optimal solution. The test results reported that the model was valid, and the assumptions of considering the stochastic and time-dependent nature of transportation networks indeed need to be highly concerned. It was not only in line with the reality, but also being regarded as an extension of the existing literature which would attract great attentions of operators and individuals who had rigid schedule time. Moreover, in future researches, the proposed model could be applied to a large-scale real transportation network to evaluate the performance and efficiency, and the uncertainties and fluctuation of travel times could be calibrated by using the float car data.

## References

[1] Miranda DM, de Camargo RS, Conceicao SV, Porto MF, Nunes NTR. A multi-loading school bus routing problem. Expert Syst Appl. 2018;101:228–42.

[2] Park J, Kim BI. The school bus routing problem: A review. Eur J Oper Res. 2010;202(2):311–9.

[3] Schittekat P, Kinable J, Sorensen K, Sevaux M, Spieksma F, Springael J. A metaheuristic for the school bus routing problem with bus stop selection. Eur J Oper Res. 2013;229(2):518–28.

[4] Toth P, Vigo D. Models, relaxations and exact approaches for the capacitated vehicle routing problem. Discrete Appl Math. 2002;123(1–3):487–512.

[5] Bektas T, Elmastas S. Solving school bus routing problems through integer programming. J Oper Res Soc. 2007;58(12):1599–604.

[6] Spada M, Bierlaire M, Liebling TM. Decision-aiding methodology for the school bus routing and scheduling problem. Transport Sci. 2005;39(4):477–90.

[7] Caceres H, Batta R, He Q. School Bus Routing with Stochastic Demand and Duration Constraints. Transport Sci. 2017;51(4):1349–64.

[8] Ellegood WA, Campbell JF, North J. Continuous approximation models for mixed load school bus routing. Transport Res B-Meth. 2015;77:182–98.

[9] Lima FMD, Pereira DSD, da Conceicao SV, de Camargo RS. A multi-objective capacitated rural school bus routing problem with heterogeneous fleet and mixed loads. 4or-Q J Oper Res. 2017;15(4):359–86.

[10] Lima FMS, Pereira DS, Conceicao SV, Nunes NTR. A mixed load capacitated rural school bus routing problem with heterogeneous fleet: Algorithms for the Brazilian context. Expert Syst Appl. 2016;56:320–34.

[11] Yao BZ, Cao QD, Wang Z, Hu P, Zhang MH, Yu B. A two-stage heuristic algorithm for the school bus routing problem with mixed load plan. Transp Lett. 2016;8(4):205–19.

[12] Prakash AA. Pruning algorithm for the least expected travel time path on stochastic and time-dependent networks. Transport Res B-Meth. 2018;108:127–47.

[13] Fugenschuh A. Solving a school bus scheduling problem with integer programming. Eur J Oper Res. 2009;193(3):867–84.

[14] Park J, Tae H, Kim BI. A post-improvement procedure for the mixed load school bus routing problem. Eur J Oper Res. 2012;217(1):204–13.

[15] Bogl M, Doerner KF, Parragh SN. The school bus routing and scheduling problem with transfers. Networks. 2015;65(2):180–203. 10.1002/net.21589 28163329 PMC5255963

[16] Riera-Ledesma J, Salazar-Gonzalez JJ. A column generation approach for a school bus routing problem with resource constraints. Comput Oper Res. 2013;40(2):566–83.

[17] Pacheco J, Caballero R, Laguna M, Molina J. Bi-objective bus routing: an application to school buses in rural areas. Transport Sci. 2013;47(3):397–411.

[18] Riera-Ledesma J, Salazar-Gonzalez JJ. Solving school bus routing using the multiple vehicle traveling purchaser problem: A branch-and-cut approach. Comput Oper Res. 2012;39(2):391–404.

[19] Rashidi TH, Zokaei-Aashtiani H, Mohammadian A. School bus routing problem in large-scale networks new approach utilizing tabu search on a case study in developing countries. Transport Res Rec. 2009;(2137):140–7.

[20] Thangiah SR, Fergany A, Wilson B, Pitluga A, Mennell W. School bus routing in rural school districts Lect Notes Econ Math. 2008;600:209–32.

[21] Ban XG, Pang JS, Liu HX, Ma R. Continuous-time point-queue models in dynamic network loading. Transport Res B-Meth. 2012;46(3):360–80.

[22] Chai H, Ma R, Zhang HM. Search for parking: a dynamic parking and route guidance system for efficient parking and traffic management. 96th Annual Meeting of the Transportation Research Board; Washington, D.C.,2017.

[23] Ma R, Ban XG, Pang JS. Continuous-time dynamic system optimum for single-destination traffic networks with queue spillbacks. Transport Res B-Meth. 2014;68:98–122.

[24] Chen P, Tong R, Lu GQ, Wang YP. The α-reliable path problem in stochastic road networks with link correlations: A moment-matching-based path finding algorithm. Expert Syst Appl. 2018;115:20–32.

[25] Sun SC, Duan ZY, Yang DY. Optimal paths planning in dynamic transportation networks with random link travel times. J Cent South Univ. 2014;21(4):1616–23.

[26] Sun SC, Duan ZY, Yang DY. Urban freight management with stochastic time-dependent travel times and application to large-scale transportation networks. Discrete Dyn Nat Soc. 2015.

